# *Tcl1* coordinately promotes metabolic shift and regulates totipotency exit

**DOI:** 10.1093/lifemedi/lnaf013

**Published:** 2025-03-14

**Authors:** Xin Gao, Chen Gao, Yikai Shi, Min Lin, Chang Du, Fei Gao, Xuguang Du, Sen Wu

**Affiliations:** State Key Laboratory of Animal Biotech Breeding, Frontiers Science Center for Molecular Design Breeding (MOE), College of Biological Sciences, China Agricultural University, Beijing 100193, China; State Key Laboratory of Animal Biotech Breeding, Frontiers Science Center for Molecular Design Breeding (MOE), College of Biological Sciences, China Agricultural University, Beijing 100193, China; State Key Laboratory of Animal Biotech Breeding, Institute of Animal Science, Chinese Academy of Agricultural Sciences, Beijing 100193, China; State Key Laboratory of Animal Biotech Breeding, Frontiers Science Center for Molecular Design Breeding (MOE), College of Biological Sciences, China Agricultural University, Beijing 100193, China; State Key Laboratory of Animal Biotech Breeding, Frontiers Science Center for Molecular Design Breeding (MOE), College of Biological Sciences, China Agricultural University, Beijing 100193, China; State Key Laboratory of Animal Biotech Breeding, Frontiers Science Center for Molecular Design Breeding (MOE), College of Biological Sciences, China Agricultural University, Beijing 100193, China; State Key Laboratory of Animal Biotech Breeding, Frontiers Science Center for Molecular Design Breeding (MOE), College of Biological Sciences, China Agricultural University, Beijing 100193, China; Sanya Institute of China Agricultural University, Sanya 572025, China; State Key Laboratory of Animal Biotech Breeding, Frontiers Science Center for Molecular Design Breeding (MOE), College of Biological Sciences, China Agricultural University, Beijing 100193, China; Sanya Institute of China Agricultural University, Sanya 572025, China; State Key Laboratory of Animal Biotech Breeding, Frontiers Science Center for Molecular Design Breeding (MOE), College of Biological Sciences, China Agricultural University, Beijing 100193, China; Sanya Institute of China Agricultural University, Sanya 572025, China

**Keywords:** totipotency, pluripotency, *Tcl1*, cell fate regulation

## Abstract

During early embryonic development, particularly in the transition from totipotency to pluripotency, energy metabolism is closely linked to cell fate. However, the essential regulators of energy metabolism in this transition remain unclear. In this study, we reveal that *Tcl1* influences energy metabolic characteristics and regulates the totipotency–pluripotency transition. Our findings demonstrate that the absence of *Tcl1* triggers the upregulation of totipotency genes and reduces H3K4me3 modifications at glycolysis enzyme promoters, thereby suppressing glycolytic processes. Furthermore, we found that a reduction in AKT, a downstream target of *Tcl1*, is associated with activation of the 2C gene and consequent shifts in energy metabolism. Specifically, AKT inhibition leads to succinate accumulation, further highlighting the role of succinate in the cell fate transition. Our findings underscore the central role of *Tcl1*–AKT–succinate axis in regulating totipotency and pluripotency through coordinated energy metabolic pathways.

## Introduction

After fertilization, mammalian embryos possess the remarkable ability to convert highly differentiated gametes into totipotent zygotes [1]. Subsequently, a series of pivotal events unfold, guiding the development of the entire organism. In the early stages, both zygotes and 2-cell embryos are regarded as totipotent cells capable of giving rise to embryonic and extra-embryonic lineages [2, 3]. Moreover, mouse embryonic stem cells (mESCs) are regarded as pluripotent cells derived from the inner cell mass (ICM) maintain the potential to differentiate into various embryonic lineages [4]. Recent studies have revealed the metastable nature of mESCs, with the ability to generate a rare subpopulation reminiscent of early embryonic cells, exhibiting 2C embryos-like characteristics [5]. This distinct subpopulation, identified through the murine endogenous retrovirus-L (MERVL), is termed as 2-cell-like cells (2CLCs). The emergence of 2CLCs presents an excellent model for investigating the molecular mechanisms regulating totipotency and ZGA *in vitro* [6, 7].

In early mammalian embryonic development, metabolic shift plays a critical role by not only regulating energy production but also driving major genomic reprogramming events [8, 9]. Recent studies have emphasized the vital role of pyruvate in initiating zygotic genome activation (ZGA), and later the early mouse embryos exclusively depending on monocarboxylates such as pyruvate and lactate for their bioenergetic needs up to the 8-cell stage [10]. Furthermore, lactate and the key metabolic cofactor nicotinamide adenine dinucleotide are implicated in regulating ZGA activation and exit by influencing epigenetic modifications in mice [11, 12]. During the morula and blastocyst stages, embryos depend on glucose for energy production and glucose-mediated signaling is crucial for the specific regulation of trophectoderm (TE) cell fate [13–15]. Consequently, as development advances from a totipotent to a more restricted pluripotent stage, there is a significant shift in carbon metabolism. The emergence of 2CLCs provides an opportunity to study the metabolic features of pre-implantation embryos [16]. Recent research has demonstrated that 2CLCs exhibit decreased glycolytic activity, akin to what is observed in 2-cell-stage embryos. This finding supports the notion of a metabolic shift occurring during the transition of mESCs to 2CLCs [17, 18]. Despite extensive investigations into the intrinsic control of embryogenesis and cell fate by metabolites, the essential regulators driving these metabolic shifts remain largely unidentified.

In our previous research, we conducted an extensive genome-wide CRISPR KO screening using a reporter mESCs line equipped with a MERVL-tdTomato reporter [19]. This screening results allowed us to uncover the intricate regulatory network orchestrating the delicate balance between totipotency and pluripotency. Upon meticulous analysis of the screening data, we pinpointed *Tcl1* as a significant candidate involved in modulating the transition toward a 2C-like state.

In this study, we identified *Tcl1* as an essential metabolic regulator for silencing 2C transcription programs. *Tcl1* demonstrates a distinct expression pattern compared to 2C genes during the transition from totipotency to pluripotency. The loss of *Tcl1* function resulted in heightened ZGA and subsequent developmental delays in mouse embryos. Our findings indicate that *Tcl1* modulates 2C gene expression through glycolysis and AKT activity, which leads to the accumulation of succinate following AKT repression. Collectively, we established a direct correlation between the *Tcl1*–AKT–succinate axis and the 2C-Like state.

## Results

### *Tcl1* influences the expression of 2C-specific genes

**Figure 1.**
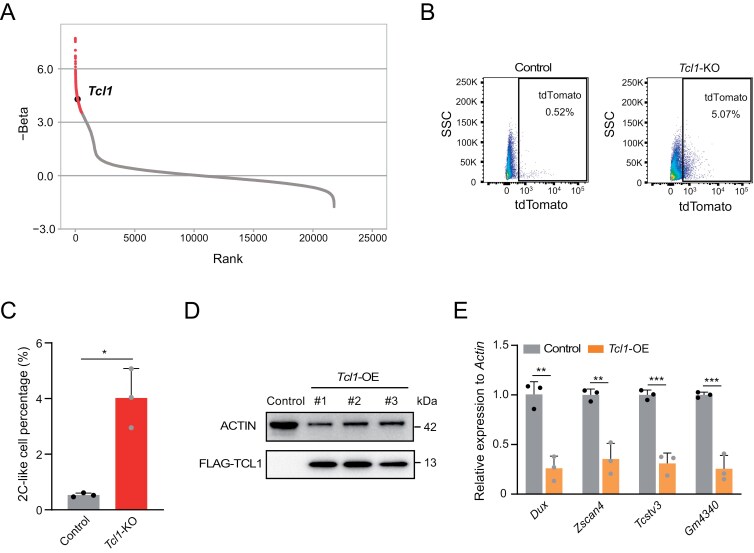
***Tcl1* absence influences metabolic shifts and activates totipotency gene transcription in mESCs.**(A) A genome-wide CRISPR/Cas9 screen identified *Tcl1* as a top candidate based on gene count enrichment. (B, C) Flow cytometry analysis was performed to assess the MERVL-tdTomato positive subpopulation in *Tcl1* KO and WT mESCs. *n* = 3 biological replicates. Data are presented as mean ± SD and analyzed using Student’s *t*-test. *P < 0.05. (D) Western blot analysis was used to examine the effects of *Tcl1* overexpression. (E) qPCR analysis of 2C gene expression levels in WT ESCs versus *Tcl1* OE mESCs, with *n* = 3 biological replicates. Data are presented as mean ± SD and analyzed using Student’s *t*-test. **P < 0.01, ***P < 0.001.

We previously conducted a genome-wide CRISPR/Cas9 screen to identify factors involved in mediating the transition of totipotency. *Tcl1* emerged as a notable candidate factor hindering this transition (Fig. 1A). To elucidate the role of *Tcl1*, we generated *Tcl1* KO mESCs using CRISPR/Cas9 technology (Fig. S1A). We observed a higher percentage of MERVL-tdTomato-positive cells in *Tcl1* KO mESCs compared to wild-type mESCs (Fig. 1B and 1C). qPCR analysis revealed increased expression of 2C markers in *Tcl1* KO mESCs (Fig. S1B). Prolonged cultivation of *Tcl1* KO mESCs sustained the presence of 2CLCs (Fig. S1C). Additionally, key pluripotency transcription factors such as *Sox2* exhibited decreased expression following *Tcl1* perturbation (Fig. S1D). Subsequently, we successfully engineered Flag-tagged *Tcl1* in mESCs (Fig. 1D). Overexpression of *Tcl1* led to a substantial downregulation of 2C genes (Fig. 1E). Taken together, these findings unequivocally demonstrate that *Tcl1* impedes the transition to the 2CLCs state in mESCs.

### *Tcl1* is down-regulated in 2C state and its perturbation results in excessive ZGA

**Figure 2.**
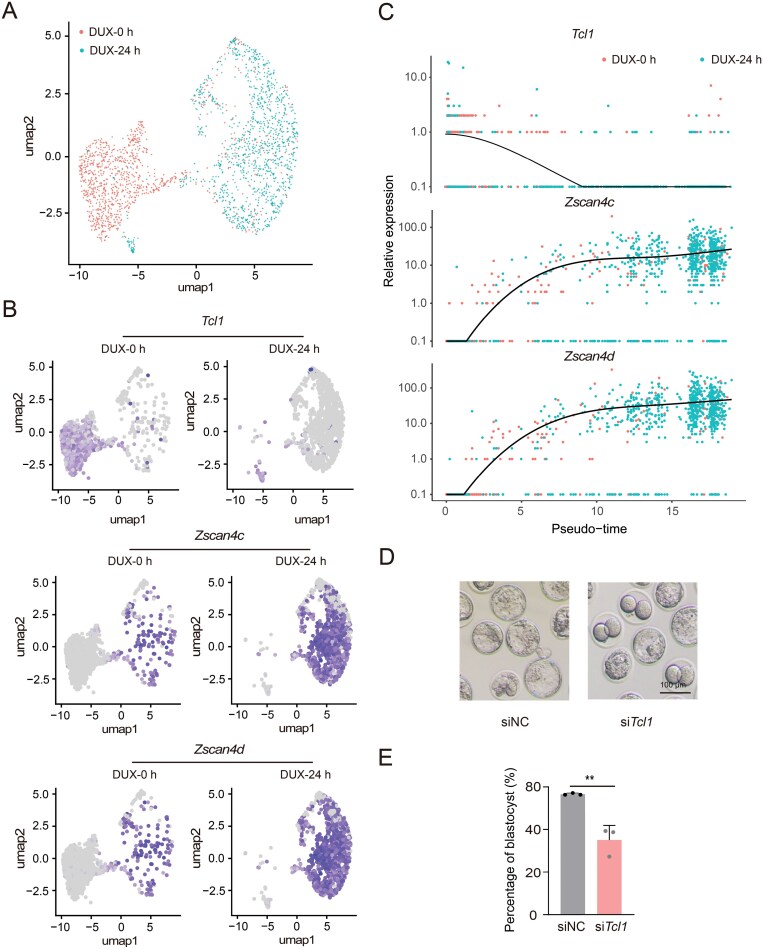
***Tcl1* perturbation activates 2C genes and arrests mouse early embryo development.** (A) UMAP plot of single-cell transcriptome data from GSE133234, illustrating the overall cell distribution. (B) UMAP plot showing expression levels of *Tcl1* and 2C genes, with gray indicating no expression and a gradient from purple to bright purple representing increasing expression levels. (C) Pseudo-time analysis revealing dynamic expression patterns of representative genes across developmental stages. (D) Representative images of blastocyst development following injection of *Tcl1* siRNA pool or control siRNA. The experiment included three biological replicates, with a scale bar of 100 μm. (E) Bar graph depicting the rates of blastocyst development after *Tcl1* siRNA pool or control siRNA injection. Data are presented as mean ± SD from *n* = 3 biological replicates and analyzed using Student's *t*-test. **P < 0.01.

We examined single-cell RNA-seq profiling data from DUX-induced 2C-like states to observe the dynamic expression patterns of *Tcl1* (Fig. 2A) [20]. Notably, *Tcl1* expression decreased upon DUX induction into the 2C-like state, showing an inverse relationship with 2C markers (Fig. 2B). Scatterplots illustrating the pseudo-time expression of *Tcl1* alongside 2C-specific genes revealed that *Tcl1* expression decreased while the expression of other 2C genes increased during the transition from pluripotency to totipotency (Fig. 2C). These findings indicated *Tcl1* was down-regulated in 2CLCs and totipotency state.

To further explore the role of *Tcl1* in totipotency regulation, we conducted RNA-seq analyses on 2CLCs derived from *Tcl1* perturbation and WT mESCs. *Tcl1* inhibition resulted in the upregulation of 3990 genes, primarily associated with RNA processing and RNA binding functions (Fig. S2A). Additionally, we noted that the perturbation of *Tcl1* in 2CLCs resulted in the upregulation of *Nr5a2*, a gene pivotal for the initiation of ZGA (Fig. S2B) [21]. Next, we performed RNA-level knockdown of *Tcl1* by injecting siRNA into zygotes to investigate the impact of *Tcl1* on totipotency (Fig. S2C). Compared to the negative control, si*Tcl1* embryos displayed delayed development and reduced entry into the morula stage at embryonic day 3.5 (Fig. S2D and S2E). Additionally, the potential for si*Tcl1* embryos to progress to the blastocyst stage was diminished, with development blocked at an earlier stage (Fig. 2D and 2E). These findings underscore that *Tcl1* perturbation causes excessive ZGA, leading to developmental delay and failure after the 2C stage.

### *Tcl1* cooperates with AKT to repress 2C genes, impeding the transition from pluripotency to totipotency.

**Figure 3.**
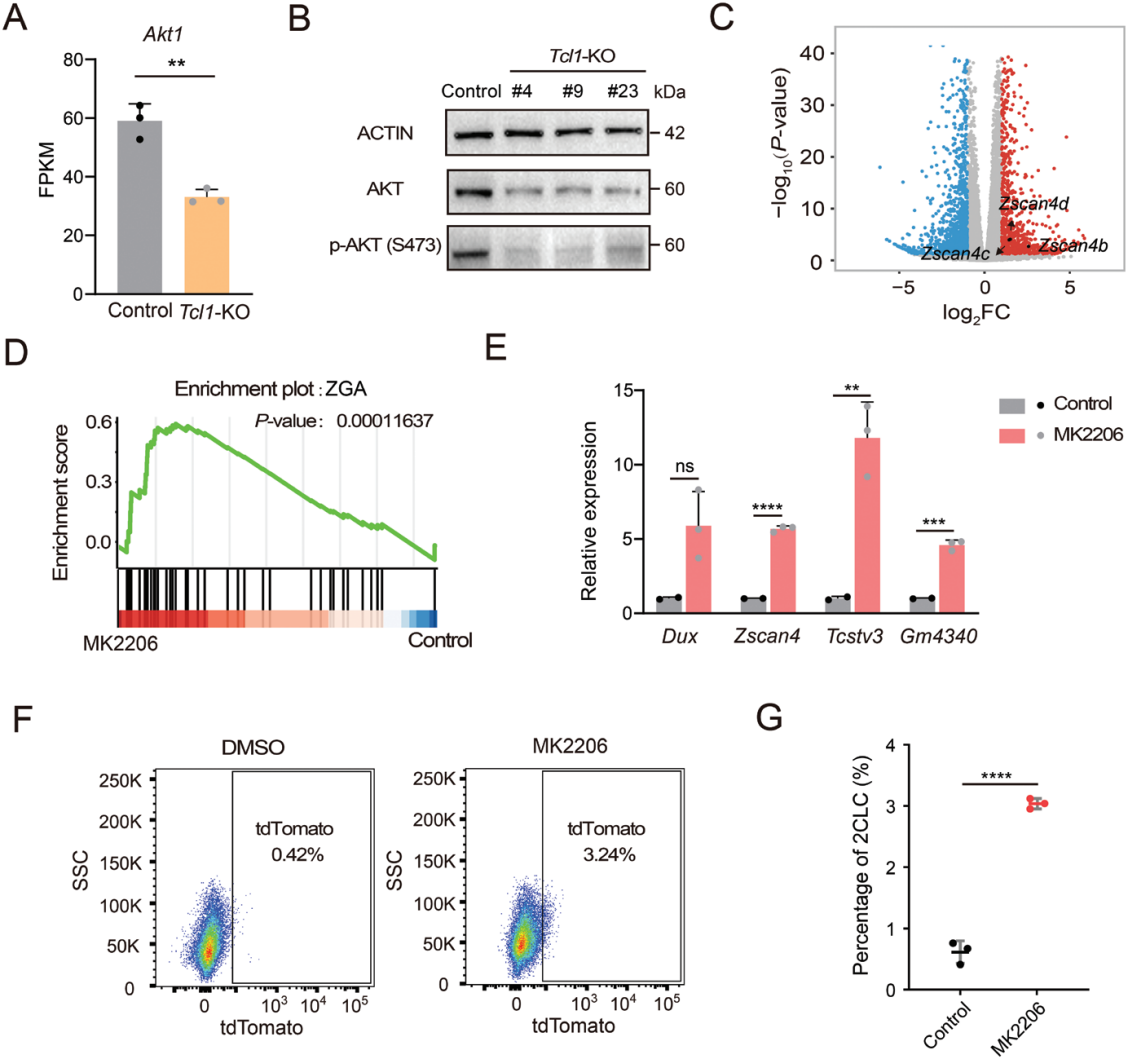
***Tcl1* cooperates with AKT to repress 2C genes.**(A) Comparison of AKT1 expression levels between WT and *Tcl1* KO mESCs. (B) Western blotting results showing p-AKT (S473) expression levels in control versus *Tcl1* KO mESCs. (C) Volcano plot visualizing DEGs in mESCs treated with DMSO versus MK2206. Genes with log_2_FC > 1 and *P* < 0.05 are shown as red dots, while genes with log_2_FC < − 1 and *P* < 0.05 are depicted as blue dots. 2C genes are explicitly labeled. (D) GSEA highlighting the upregulation of the ZGA pathway in mESCs treated with MK2206 compared to DMSO. (E) qPCR analysis of totipotency gene expression levels in mESCs treated with DMSO versus MK2206. (F, G) Flow cytometry analysis assessing the MERVL-tdTomato positive subpopulation in mESCs treated with DMSO and MK2206. *n* = 3 biological replicates. Data are presented as mean ± SD, analyzed using Student’s *t*-test. *P < 0.05, **P < 0.01, ***P < 0.001, ****P < 0.0001.

It was previously reported *Tcl1* modulated AKT activity which acts as a coactivator to enhance AKT kinase activity [22]. Hence, we investigated AKT alterations in *Tcl1* KO mESCs, finding a significant decrease in both AKT expression and its phosphorylated, active form at Ser473 (Fig. 3A and 3B). Consistently, *Tcl1* OE in mESCs increased levels of phosphorylated AKT (Fig. S3A). To determine if AKT influenced the 2C state, we used MK2206, a selective AKT inhibitor, to evaluate this potential effect [23]. Treatment with MK2206 markedly reduced phosphorylated AKT and total AKT expression, consistent with previous studies (Fig. S3B). Consistent with previous reports, AKT suppression also resulted in decreased cellular proliferation rates and notable changes in the cell cycle, notably an increase in the G1 phase (Fig. S3C and S3D) [24]. Interestingly, AKT expression was reduced following *Dux* induction into the 2C-like state, aligning with the expression pattern of *Tcl1* (Fig. S3E). RNA-seq analysis of MK2206-treated mESCs revealed significant upregulation of 2C markers, particularly within the *Zscan4* cluster (Fig. 3C). Gene Set Enrichment Analysis (GSEA) further underscored the substantial upregulation of the ZGA gene set following AKT inhibition (Fig. 3D). qPCR results supported increased expression of 2C genes following AKT suppression (Fig. 3E). Additionally, MK2206 treatment of a 2-cell-reporter mESCs line, characterized by MERVL expression, showed an increased proportion of 2CLCs (Fig. 3F and 3G). Furthermore, rescue of 2C gene expression was observed after MK2206 treatment in *Tcl1* OE mESCs (Fig. S3F). Together, these findings provide evidence that AKT, as a downstream target of *Tcl1*, acts to repress the 2C transcriptional program in mESCs.

### Zygotic repression of AKT compromises the transition from totipotency to pluripotency

**Figure 4.**
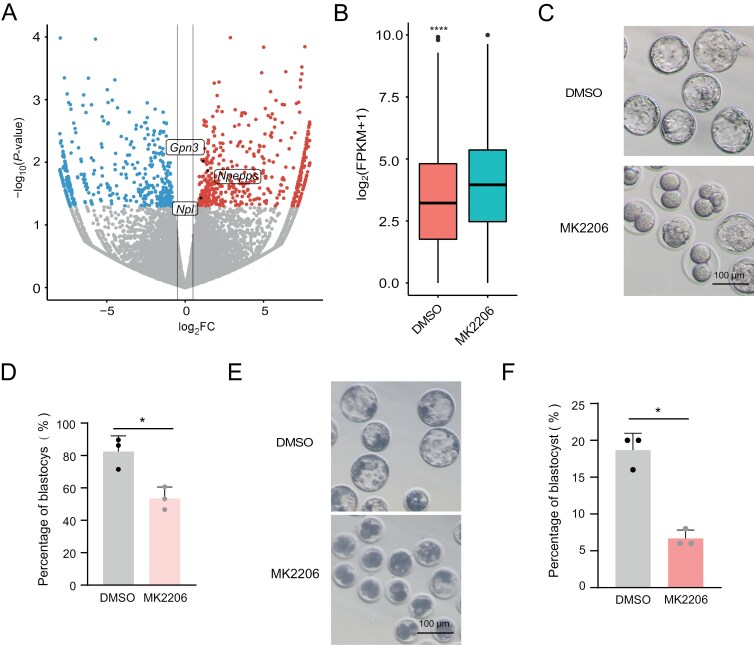
**Zygotic repression of AKT compromises the totipotency-to-pluripotency transition.**(A) Volcano plot showing DEGs in late 2C stage embryos treated with DMSO versus MK2206. Genes with log_2_FC > 0.5 and *P* < 0.05 are indicated as red dots, while genes with log_2_FC < −0.5 and *P* < 0.05 are shown as blue dots. 2C genes are explicitly labeled. (B) Expression levels of ZGA genes in late 2C embryos treated with DMSO versus MK2206. (C) Representative images of blastocyst development following MK2206 and DMSO treatment. The experiment was conducted with three biological replicates. Scale bar = 100 μm. (D) Bar graph depicting blastocyst development rates after MK2206 or DMSO treatment. (E) Representative images of blastocyst development in pig embryos following MK2206 treatment. The experiment was conducted with three biological replicates. Scale bar = 100 μm. (F) Bar plot showing the blastocyst formation rate of pig embryos after MK2206 treatment. *n* = 3 biological replicates. Data are presented as mean ± SD and analyzed using Student’s *t*-test. *P < 0.05, **P < 0.01.

To assess the role of AKT activity in totipotency regulation, we treated zygotes with MK2206 and monitored its impact on development. Initially, RNA-seq analysis of late 2C embryos treated with MK2206 compared to untreated controls revealed significant changes in gene expression. Following MK2206 treatment, 512 genes were upregulated, while 501 genes were downregulated (Fig. 4A). Importantly, among the upregulated genes, 51 were rigorously defined as ZGA genes, contrasting with only 10 among the downregulated genes (Figs. 4A and S4A). This indicated that AKT primarily opposes the upregulation of ZGA genes. Furthermore, we observed a significant increase in the expression of ZGA genes after MK2206 treatment (Fig. 4B). These findings underscored that repression of AKT results in significant upregulation of ZGA genes during the late 2-cell stages.

Moreover, developmental assessment of embryos after MK2206 treatment mirrored observations from *Tcl1* knockdown experiments, showing developmental arrest and reduced formation of blastocysts (Figs. 4C, 4D, S4B and S4C). Recognizing the critical role of AKT in ZGA regulation and mouse embryonic development, we investigated whether a similar mechanism exists in porcine embryos. As expected, MK2206 treatment reduced the rate of blastocyst development in the porcine group, with more embryos arrested at early stages (Figs. 4E, 4F and S4D). In conclusion, AKT repression leads to excessive activation of ZGA genes, potentially resulting in developmental arrest in both murine and porcine embryos.

### *Tcl1* inhibits totipotency by activating AKT and sustaining glycolytic flux

**Figure 5.**
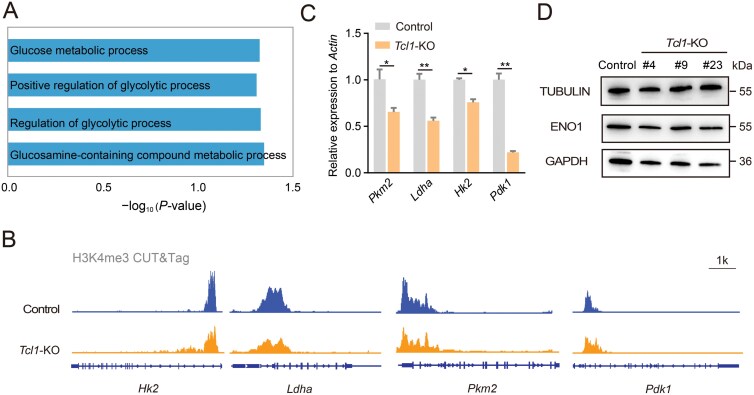
***Tcl1* influences metabolic shifts, altering cell fate.**(A) GO analysis was conducted to investigate metabolic pathways in *Tcl1* KO mESCs compared to WT mESCs. (B) IGV visualization of H3K4me3 modifications for glycolysis genes in *Tcl1* KO and WT mESCs. (C) qPCR analysis assessed the expression levels of glycolysis genes in WT versus *Tcl1* KO mESCs. *n* = 3 biological replicates. Data are presented as mean ± SD and analyzed using Student’s *t*-test. *P < 0.05, **P < 0.01. (D) The protein levels of genes associated with glycolysis were examined in *Tcl1* KO and wild-type mESCs.

To elucidate the impact of *Tcl1* perturbation on biological processes, we conducted a comprehensive analysis of GO database. This analysis revealed a downregulation across multiple metabolic pathways, including glycolytic process (Fig. 5A). Glycolysis, a pivotal metabolic pathway, has been observed to induce 2CLCs after inhibition, as demonstrated by the use of compounds like 2-deoxy-D-glucose [17]. Our previous genome-wide CRISPR/Cas9 screen also revealed significant enrichment of the glycolysis pathway (Fig. S5A) [19]. Notably, *Eno3* and *Gck* emerged as highly ranked candidates, with *Eno3* previously identified as a key inhibitor of the 2C-like state (Fig. S5B). When comparing the expression levels of glycolytic genes between ESCs and 2CLCs, we observed an overall decrease in their expression in 2CLCs (Fig. S5C). Given the potential impact of *Tcl1* on metabolism, we investigated whether *Tcl1* perturbation affected glycolysis. Remarkably, *Tcl1* depletion resulted in a concurrent decrease in H3K4me3 modification at several genes pivotal for glucose metabolism, such as *Hk2*, *Pkm2*, *Pdk1*, and *Ldha* (Fig. 5B). Both protein and mRNA levels validated the downregulation of these crucial glycolytic enzymes (Fig. 5C and 5D). Overall, our results support the notion that *Tcl1* depletion could lead to decreases in H3K4me3 modification, thereby repressing glycolysis.

**Figure 6.**
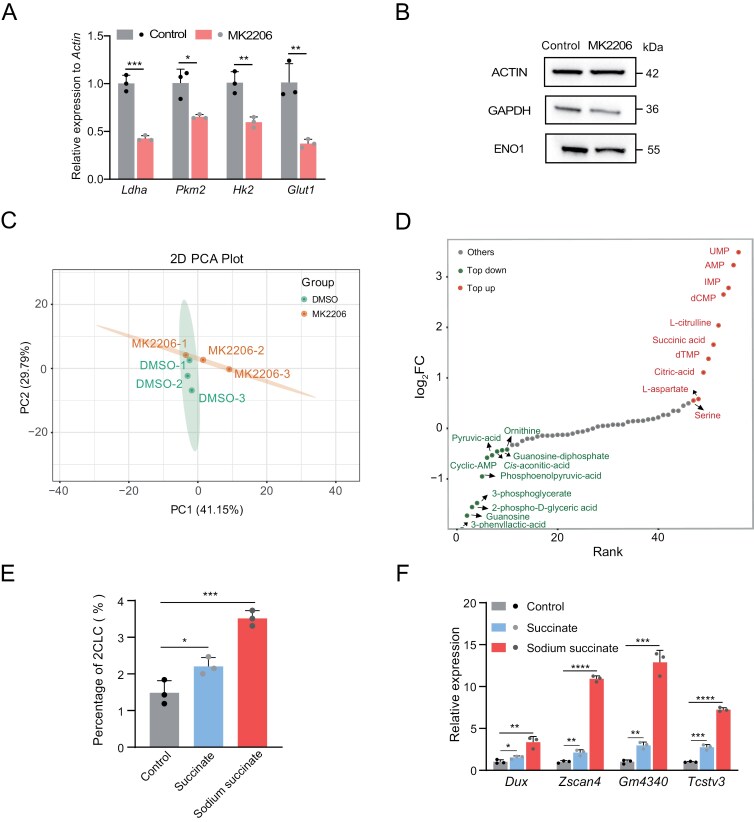
**AKT as the downstream of *Tcl1* influence glycolysis activity and cause metabolites alteration.**(A) mRNA levels of glycolysis-related genes in mESCs treated with MK2206 compared to DMSO. (B) Western blot analysis showing expression levels of glycolysis-related proteins in mESCs treated with DMSO versus MK2206. (C) Principal Component Analysis (PCA) of metabolome data from mESCs treated with DMSO and MK2206. (D) Dynamic distribution map of differentially metabolites in mESCs treated with MK2206 compared to DMSO. Red dots represent metabolites with log_2_FC > 0.5, while green dots represent those with log_2_FC < −0.5. The analysis includes three biological replicates per group (E) MERVL-tdTomato positive subpopulation in the mESCs with 5 mM succinate and 60 mM sodium succinate. (F) qPCR analysis was conducted to assess the expression levels of totipotency genes in WT ESCs compared to the mESCs with 5 mM succinate and 60 mM sodium succinate. *n* = 3 biological replicates. Data are presented as mean ± SD and analyzed using Student’s *t*-test. *P < 0.05, **P < 0.01.

Furthermore, our study delineated that treatment with MK2206, significantly attenuated glycolysis, as evidenced by reductions in both transcript and protein levels (Fig. 6A and 6B). This effect mirrors the observed phenomenon following *Tcl1* depletion. Additionally, we performed targeted metabolomic analysis in MK2206-treated cells (Fig. 6C). It revealed a significant decrease in glycolytic intermediates (Fig. 6D). Moreover, tricarboxylic acid cycle (TCA) cycle intermediates, such as citric acid and succinate, were predominantly upregulated in MK2206-treated cells (Fig. 6D). To further understand the broader metabolic ramifications, we treated MERVL reporter mESCs with sodium succinate and succinate, and observed the expansion of the 2CLCs population and activation of the 2C program (Fig. 6E and 6F). Together, these findings provide compelling evidence that *Tcl1* inhibited the 2C transcriptional program, through influencing AKT activity and metabolic shift and suggested the critical roles of the *Tcl1*–AKT–succinate axis in regulating the 2C state.

## Discussion

**Figure 7.**
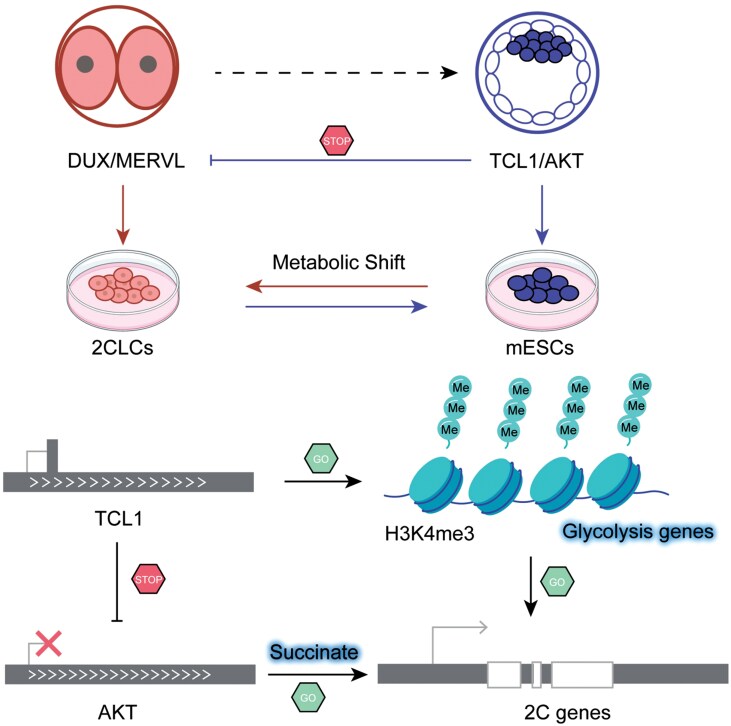
***Tcl1* impedes 2C genes transcription in mESCs.**Schematic model illustrating the mechanism by which *Tcl1* inhibits 2C genes transcription in mESCs.

In this study, we elucidate the critical role of *Tcl1* in the transition from totipotency to pluripotency during early embryonic development and in mESCs. Our findings indicate that *Tcl1* deficiency leads to the high expression of genes associated with the 2C state and a decrease in glycolysis activity. Furthermore, the depletion of *Tcl1* results in the repression of AKT, causing a decrease in glycolysis intermediates and an increase in TCA cycle intermediates, particularly succinate, which promotes the 2C-like state (Fig. 7). Specifically, we identify a novel *Tcl1*–AKT–succinate pathway that regulates the transitions between totipotency and pluripotency.

*Tcl1* is prominently expressed in mESCs and serves as a crucial gene for maintaining self-renewal and pluripotency [25–28]. Mice homozygous for the *Tcl1* knockout allele display maternal fertility defects, resulting in a shortened reproductive lifespan and a gradual decrease in offspring [29]. This indicates the essential role of *Tcl1* in early embryonic development. Here, we found the distinct expression pattern of *Tcl1* compared to ZGA genes during the transition from totipotency to pluripotency underscores its unique regulatory function. Inhibition of *Tcl1* caused heightened ZGA and developmental delays in mouse embryos, indicating its crucial role in embryogenesis progression. These results suggest that *Tcl1* is indispensable for the proper regulation of gene expression during early development, highlighting its potential as a target for enhancing fertility and addressing developmental disorders.

2CLCs exhibit unique metabolic characteristics in contrast to ESCs, characterized by lower glycolytic and respiratory activity [17, 18, 30]. Manipulating specific energy metabolites might induce totipotent states [17, 18]. This metabolic distinction prompted us to investigate the involvement of significant metabolic regulators in the transition to the 2C-like state. Here, we identified *Tcl1* as a critical regulator of the metabolic shift during the transition from totipotency to pluripotency. *Tcl1* knockout resulted in decreased H3K4me3 modifications specifically at glycolytic gene promoters and inhibited glycolysis pathway. Given that H3K4me3 plays an essential role in transcriptional regulation during early embryonic development, including the activation of genes vital for the first cell fate determination post-ZGA [31], this observation suggests a link between *Tcl1*-mediated metabolic regulation and epigenetic modifications.

During early embryonic development, a subset of TCA cycle enzymes, such as *Pdha1*, briefly localize to and are active within the nucleus of early-stage embryos, where their metabolites play a role in epigenetic remodeling during ZGA and developmental progression beyond the 2-cell stage [10, 32]. *Pdha1*, in particular, facilitates the 2-cell program in pluripotent stem cells, indicating that TCA cycle enzymes and their metabolites are essential for epigenetic control and zygotic genome activation [32]. Recently, it has been shown that lactate-mediated epigenetic remodeling functions during ZGA [12]. Our study demonstrated that succinate accumulation facilitates the 2-cell program in mESCs. Notably, sodium succinate consistently enhanced the expression of 2C-associated genes in both *Tcl1* knockout and overexpression mESCs, suggesting that its role in promoting the 2C state involves multiple pathways beyond the *Tcl1*–AKT axis, with *Tcl1*–AKT signaling as one such mechanism (Fig. S5D). Succinylation, a post-translational protein acylation modification, alters the lysine side chain from a cationic to an anionic state, inducing substantial changes in protein structure and function [33]. A critical avenue for future research is to elucidate whether succinylation directly contributes to ZGA in both *in vivo* and *in vitro* contexts, with particular emphasis on uncovering the precise role of succinate in regulating these processes [34]. These results highlight that metabolic intermediates could serve as key regulators of developmental processes and potential targets for enhancing stem cell reprogramming and differentiation.

The identification of the *Tcl1*–AKT–succinate pathway enhances our understanding of in embryonic development and stem cell biology. Future investigations into the precise molecular mechanisms underlying *Tcl1*-mediated transitions between totipotency and pluripotency will provide deeper insights into developmental biology and potential therapeutic strategies for regenerative medicine.

## Research limitations

This study predominantly utilizes mESCs and 2CLCs as a model and the reliance on *in vitro* systems may overlook complexity of *in vivo* development. Furthermore, the generalizability of these findings to other species or developmental contexts requires further experimental validation.

## Methods

### Animal

The mice were kept at the China Agricultural University Laboratory Animals Resource Center, following a 12-hour light–dark cycle and a maintained temperature of 20°C–22°C.

### Research ethic

All animal procedures were approved by the Animal Welfare Committee of China Agricultural University (Approval No. AW03111202-3-1). ICR mice were obtained from Beijing Vital River Laboratory Animal Technology.

### Cell culture

mESCs were cultured in 500 mL KnockOut DMEM (Gibco, 10829018) with 15% FBS (Gibco, 10099), 5 mL GlutaMAX (Gibco, 35050079), 5 mL non-essential amino acids (Gibco, 11140050), 5 mL penicillin/streptomycin (Gibco, 15104122), and 100 µM 2-mercaptoethanol (Gibco, 15104122), supplemented with 50 µL LIF (Millipore, ESG1106). Feeder cells were maintained in 500 mL DMEM (Gibco, 11960) with 10% FBS (Gibco, 10099), 5 mL non-essential amino acids (Gibco, 11140050), and 5 mL penicillin/streptomycin. In the MK2206 (Selleck, S1078) experiments, ESCs were exposed to 1 uM MK2206 for up to 7 days. mESCs were passaged at a ratio ranging from 1:6 to 1:10.

### Generation of CRISPR KO mESCs lines

We employed CHOPCHOP for sgRNA design and utilize two sgRNAs and Cas9 to edit *Tcl1*. The sgRNAs were incorporated into the pSg6 plasmid. A total of 10^6^ mESCs were transfected using the Lonza 2B Nucleofector system. After 24 h, transfected cells were enriched with 350 ng/µL G418 (InvivoGen, ant-gn-5). Following selection, individual clones were isolated for gene type identification. Details of the sgRNAs used are provided in Table S1.

### Generation of Tcl1 overexpress mESCs lines

We generated mESCs lines overexpressing *Tcl1* by amplifying mouse cDNA. Subsequently, these fragments were constructed into the PB vector, with the EF1α promoter. The vectors were transfected into cells using the Lonza 2B Nucleofector system with the B016 program. Twenty-four hours post-transfection, cells were enriched using 350 ng/µL G418. Following selection, individual clones were isolated for assessing expression levels.

### siRNA transfection

We synthesized SiRNAs at Sangon and used Lipofectamine 2000 to transfect mESCs (Thermo Fisher Scientific, 11668030), following Sangon’s instructions. The evaluation of knockdown effects was conducted 72 h after transfection. Comprehensive information regarding the siRNAs is available in Table S1.

### Embryo collection

ICR mice were administered injections of pregnant mare serum gonadotropin and human chorionic gonadotropin 46 h apart. Subsequently, embryos were collected at specified intervals post-hCG injection, with time points including 24 h for the zygote stage, 58 h for the 4C embryo, 69 h for the 8C embryo.

### Embryo injection

We injected zygotes with a solution containing 50 μM siRNA, with each zygote receiving approximately 5 pL of the injection. Subsequently, the injected embryos were cultured at 37°C with 5% CO_2_. Continuous observations were conducted daily, extending up to 4.5 days.

### Quantitative reverse-transcription PCR

Total RNA was extracted using RaPure Total RNA Kits (Magene, R4011). One microgram of RNA was reverse-transcribed into cDNA using the ABScript III RT Master Mix (Abclonal, RK20429). The resulting cDNA was diluted tenfold and subjected to amplification with 2 × RealStar Green Power Mix (Genestar, A311-10). Actin served as the reference gene for normalization. Details of the primers used for qPCR are provided in Table S1.

### Fluorescence-activated cell sorting (FACS)

The expression of MERVL was examined utilizing the FACS CaliburTM flow cytometer (BD, San Jose, CA, USA), and the acquired data were visualized employing FlowJo software, version 10.

### Cell cycle analysis

A total of 1 × 10^6^ cells were subjected to fixation in 70% ethanol at − 20°C for a duration of 4 h. Subsequently, the cell cycle assay kit (Elabscience, E-CK-A351) was employed to assess alterations in the cell cycle, following the manufacturer’s specified protocols. Flow cytometry was utilized to measure DNA content, and the resulting data were analyzed using Modfit LT software.

### Western blot

A total of 1 × 10^6^ cells were lysed using 200 µL of immunoprecipitation (IP) lysis buffer and incubated on ice for 30 min. Subsequently, the supernatant was collected by centrifugation at 23,000 ×*g* for 10 min at 4°C. Protein concentration was determined using the BCA protein assay kit (Beyotime, P0012). An appropriate volume of the supernatant was mixed with 10% SDS–PAGE and denatured at 95°C for 10 min. For Western blot, primary antibodies including ACTIN (Beyotime, AA128), ENO1 (Abclonal, A16841), GAPDH (Cell Signal Technology, 14C10), AKT (Abmart, T55561), and Phospho-Akt (Ser473) (Abmart, T40067) were utilized.

### RNA-seq and bioinformatics analysis

Total RNA was isolated and evaluated utilizing the RNAprep Pure Cell/Bacteria Kit (TIANGEN) and the Agilent 2100 BioAnalyzer in accordance with the manufacturer’s protocols. Following RNA extraction, the RNA library was gained employing the NEBNext Ultra^TM^ RNA Library Prep Kit and subsequently subjected to paired-end 150 base pair sequencing on an Illumina platform. Clean reads were aligned to the mm10 reference genome using HISAT2. Counts were computed and normalized to fragments per kilobase of transcript per million mapped reads (FPKM). DESeq2 was employed for the analysis of differentially expressed genes. Further analyses involving pathway analysis and data interpretation were conducted using the R programming language.

### CUT&Tag

The generation of the CUT&Tag library was accomplished utilizing the NovoNGS CUT&Tag 3.0 High-Sensitivity Kit (Novoprotein, N259). In summary, 10^5^ cells were captured using 10 μL of Binding ConA beads. Primary antibodies specific to H3K4me3 (Proteintech, 39160) were employed to incubate the cell-bead complexes for 2 h at room temperature, followed by the addition of secondary antibodies. After incubation, Transposome was introduced into the mixture, and the transposition reaction was conducted at 37°C for 1 h. Following the transposition reaction, DNA extraction was carried out and subsequently purified employing extraction beads.

### CUT&Tag-seq analysis

High-quality reads were aligned to the mm10 reference genome using bowtie2 and default parameters. Subsequently, the resultant aligned reads were transformed into BAM files format through the utilization of Samtools. The peak calling was performed using MACS2. The identified peaks were subjected to analysis and annotation through the use of ChIPseeker and Deeptools.

### Public datasets reanalyzed

We obtained the transcriptome data for mouse pre-implantation embryos from the GSE71434 dataset and single-cell transcriptome of mESCs from GSE133234.

### Statistical analysis

The results underwent analysis employing the Student’s *t*-test or two-way ANOVA test, with statistical evaluations executed using GraphPad Prism. Significance levels were indicated as follows: *P < 0.05, **P < 0.01, ***P < 0.001, ****P < 0.0001.

## Supplementary Material

lnaf013_suppl_Supplementary_Figures_S1-S5_Table_S1

## Data Availability

The datasets produced and analyzed during this study are available from the corresponding author on reasonable request.
