# Supplementary material for: Tcl1 coordinately promotes metabolic shift and regulates totipotency exit
Source: Life Med. 2025 Mar 14;4(3):lnaf013. doi: 10.1093/lifemedi/lnaf013 (PMC12076405; doi:10.1093/lifemedi/lnaf013)
Supplement: lnaf013_suppl_Supplementary_Figures_S1-S5_Table_S1 [file lnaf013_suppl_supplementary_figures_s1-s5_table_s1.zip › Supplemental_Figure_legends_PE.docx]

***Tcl1* coordinately promotes metabolic shift and regulates totipotency exit**

Xin Gao^1,#^, Chen Gao^1,2,#^, Yikai Shi^1^, Min Lin^1^, Chang Du^1^, Fei Gao^1,3^, Xuguang Du^1,3,*^, Sen Wu^1,3,*^

^1^State Key Laboratory of Animal Biotech Breeding, Frontiers Science Center for Molecular Design Breeding (MOE), College of Biological Sciences, China Agricultural University, Beijing 100193, China

^2^State Key Laboratory of Animal Biotech Breeding, Institute of Animal Science, Chinese Academy of Agricultural Sciences, Beijing 100193, China

^3^Sanya Institute of China Agricultural University, Sanya 572025, China

^#^These authors contributed equally to this work.

^*^Correspondence: swu@cau.edu.cn (S.W.), xuguangdu@cau.edu.cn (X.D.)

**Supplemental figures**

**Figure S1. The characterization of mESCs with *Tcl1* KO and OE.**

(A) Schematic representation of the *Tcl1* knockout genome sequence, showing the sgRNA target sites used for introducing mutations, indicated by arrowheads. The numbers denote the base pair deletions and insertions. (B) qPCR analysis was conducted to assess the expression levels of totipotency genes in WT ESCs compared to *Tcl1* KO mESCs. (C) The percentage of 2CLCs was monitored over time following *Tcl1* KO. (D) Protein levels of *Sox2* in *Tcl1* KO mESCs were assessed. Data are presented as mean ± SD and analyzed using Student’s *t*-test. ***P* < 0.01, ****P* < 0.001, *****P* < 0.0001.

**Figure S2. Knockdown of *Tcl1* hinders early mouse embryo development.**

(A) Gene Ontology analysis of upregulated genes affected by *Tcl1* perturbation in 2CLCs. (B) Comparison of *Nr5a2* expression levels between WT and *Tcl1* perturbation 2CLCs. *n* = 3 biological replicates. Data are presented as mean ± SD and analyzed using Student’s *t*-test. ***P* < 0.01. (C) Schematic representation of the experimental procedure involving siRNA microinjection. (D, E) Imaging and quantification of mouse embryos at various stages post-injection of *Tcl1* siRNA pool or control siRNA. The study includes three biological replicates, with a scale bar set at 100 μm.

**Figure S3. The characterization of mESCs treated with MK2206.**

(A) Western blot analysis of p-AKT (S473) expression levels in control versus *Tcl1* OE mESCs. (B) Western blot analysis showing expression levels of AKT and p-AKT (S473) in mESCs treated with DMSO versus MK2206. (C) Growth curves for mESCs treated with DMSO or MK2206. Data are presented as mean ± SD and analyzed using Student’s *t*-test. (D) FACS analysis of cell cycle phase distribution in mESCs following MK2206 treatment, based on three independent experiments. Data are presented as mean ± SD and analyzed using Student’s *t*-test. (E) The violin plot showing the expression levels of AKT genes. (F) qPCR analysis of 2C gene expression levels in *Tcl1* KO and OE mESCs treated with DMSO versus MK2206. Data are presented as mean ± SD and analyzed using two-way ANOVA test. **P* < 0.05, ***P* < 0.01, ****P* < 0.001, *****P* < 0.0001.

**Figure S4. Repression of AKT activity affects the development of early mouse embryos.**

(A) Ring plots illustrating the proportion of DEGs in the late 2C stage following MK2206 treatment. (B, C) Mouse embryos at various developmental stages imaged and counted to assess developmental progress after DMSO or MK2206 treatment. The study includes three biological replicates, with a scale bar of 100 μm. (D) Developmental progression of pig embryos at different stages following DMSO or MK2206 treatment on day 7.

**Figure S5. Analysis of the results of CRISPR knockout screening.**

(A) KEGG analysis highlighting the enrichment of metabolic pathways identified in the screening. (B) Analysis of enriched genes within the glycolysis pathway. (C) Bar plots displaying the expression levels of glycolysis genes in 2CLCs compared to mESCs. *n* = 3 biological replicates. Data are presented as mean ± SD and analyzed using Student’s *t*-test. **P* < 0.05, ***P* < 0.01. (D) qPCR analysis of 2C gene expression levels in *Tcl1* KO and OE mESCs treated with Na succinate. Data are presented as mean ± SD and analyzed using two-way ANOVA test. *****P* < 0.0001.
