# Supplementary figures and images for: Tcl1 coordinately promotes metabolic shift and regulates totipotency exit
Source: Life Med. 2025 Mar 14;4(3):lnaf013. doi: 10.1093/lifemedi/lnaf013 (PMC12076405; doi:10.1093/lifemedi/lnaf013)

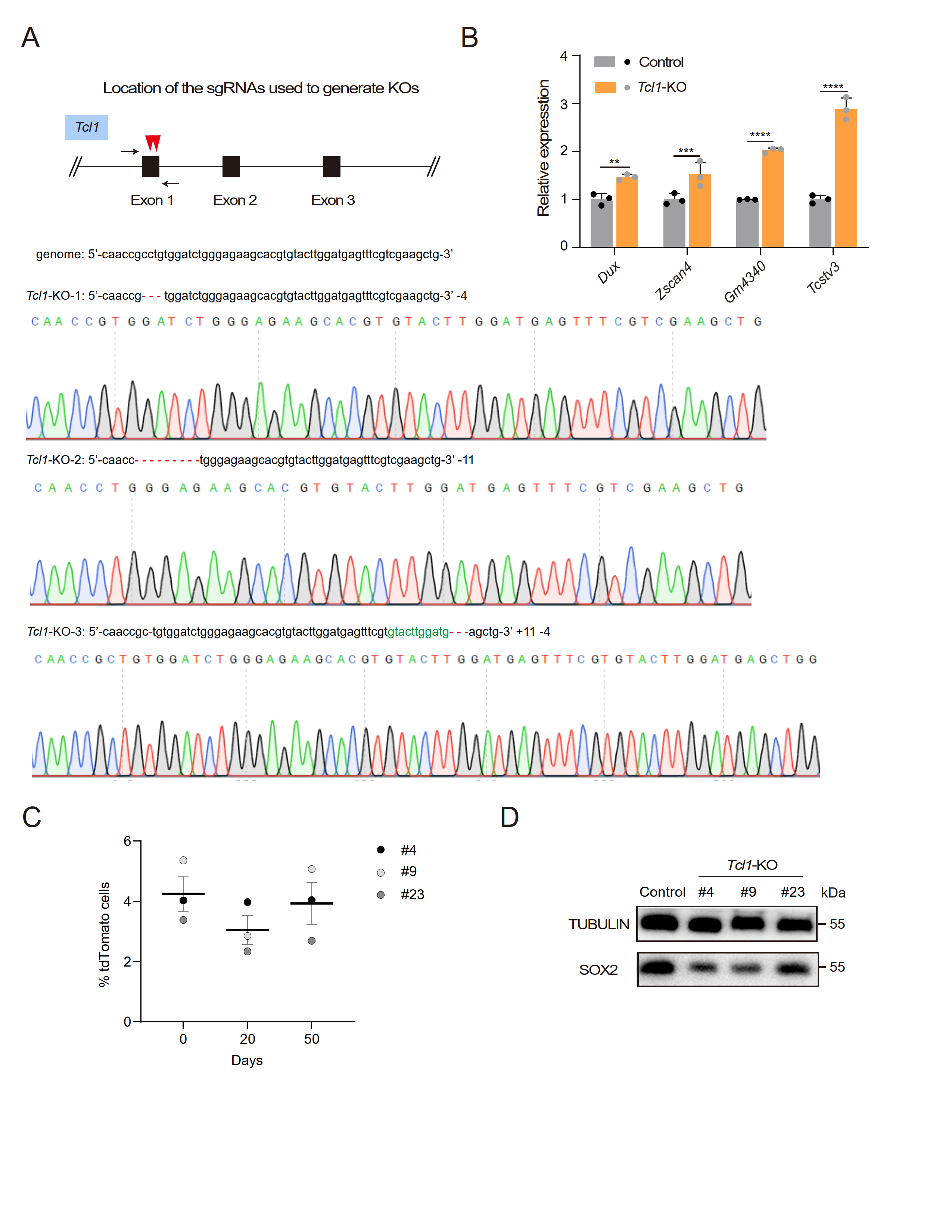

Supplement: lnaf013_suppl_Supplementary_Figures_S1-S5_Table_S1 [file lnaf013_suppl_supplementary_figures_s1-s5_table_s1.zip › Supplemental_Fig_S1.tif]

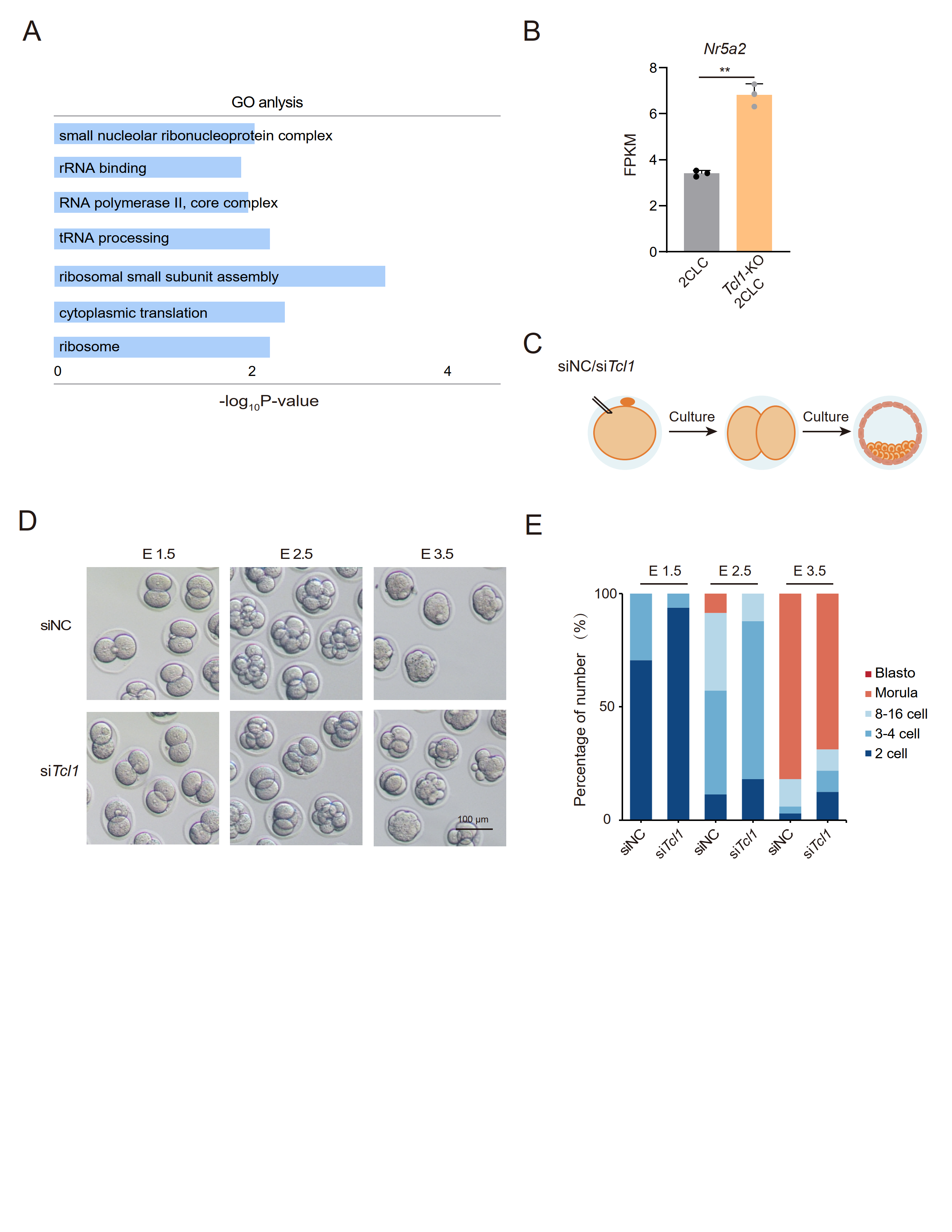

Supplement: lnaf013_suppl_Supplementary_Figures_S1-S5_Table_S1 [file lnaf013_suppl_supplementary_figures_s1-s5_table_s1.zip › Supplemental_Fig_S2.tif]

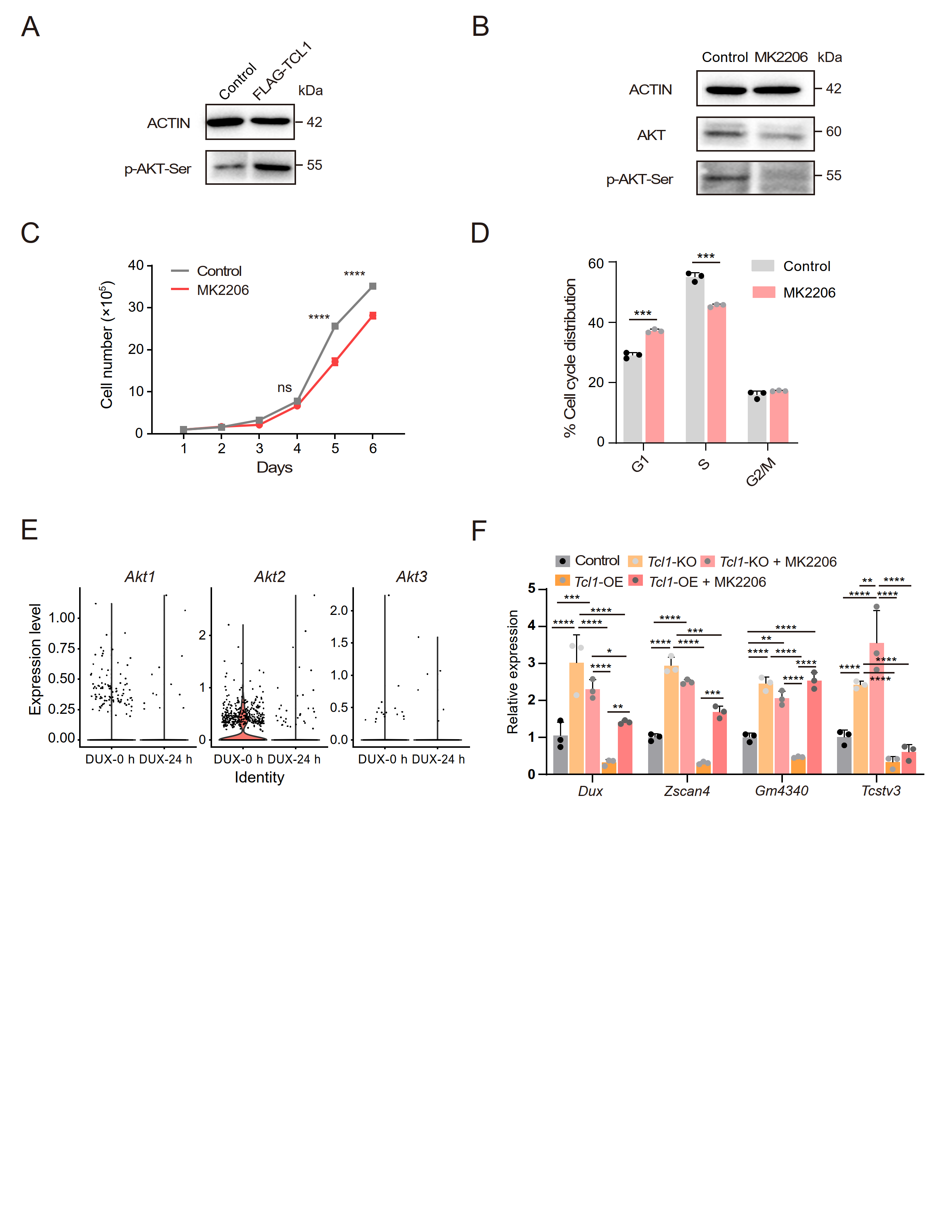

Supplement: lnaf013_suppl_Supplementary_Figures_S1-S5_Table_S1 [file lnaf013_suppl_supplementary_figures_s1-s5_table_s1.zip › Supplemental_Fig_S3.tif]

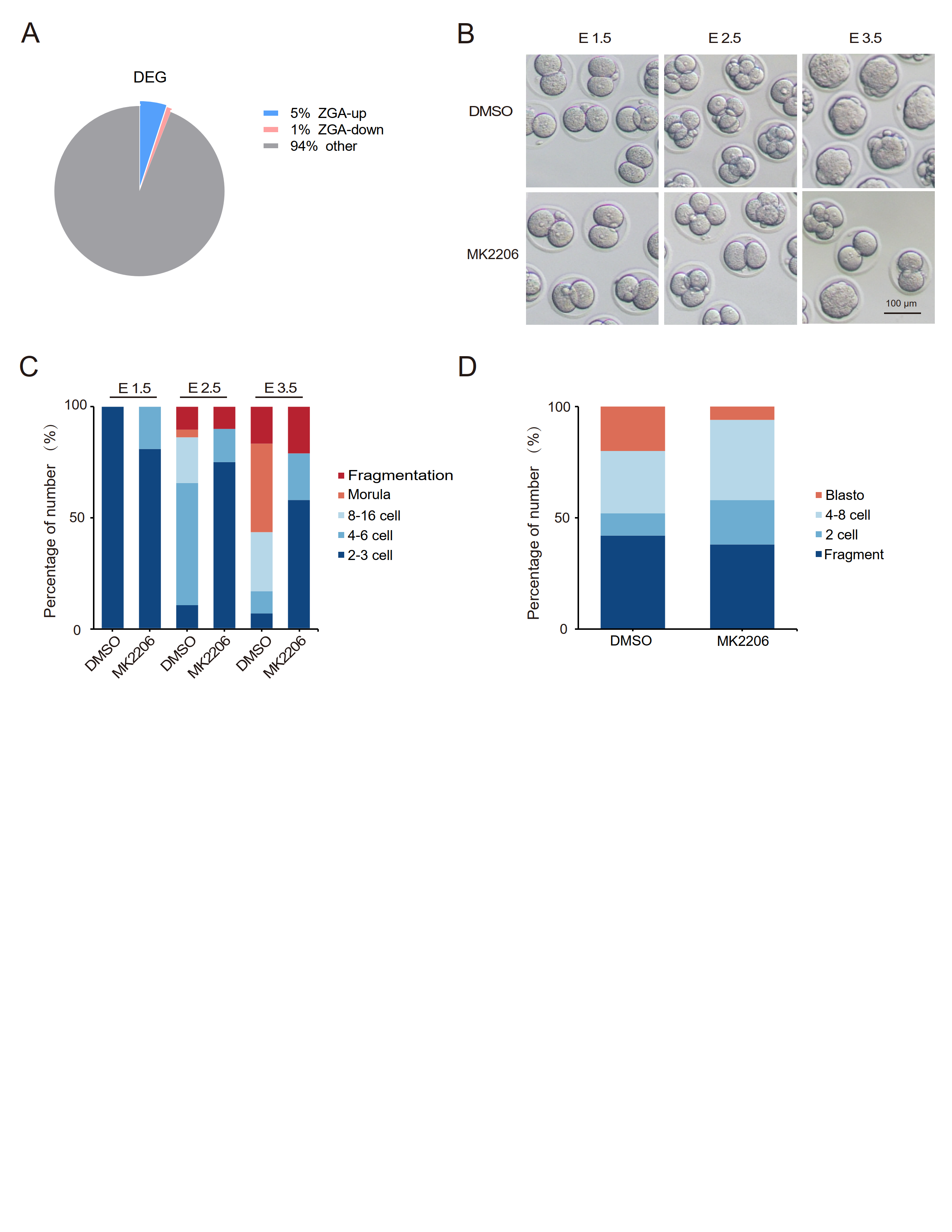

Supplement: lnaf013_suppl_Supplementary_Figures_S1-S5_Table_S1 [file lnaf013_suppl_supplementary_figures_s1-s5_table_s1.zip › Supplemental_Fig_S4.tif]

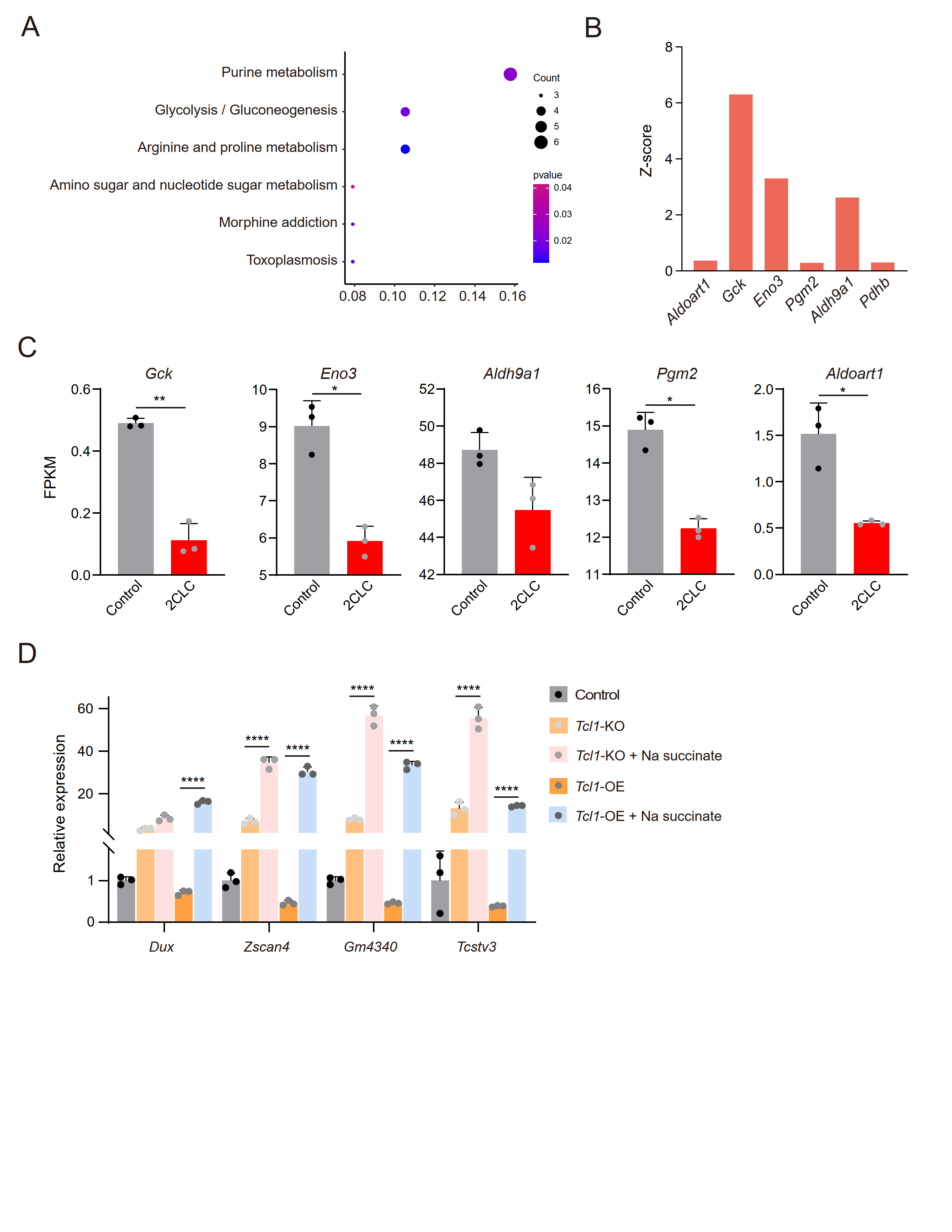

Supplement: lnaf013_suppl_Supplementary_Figures_S1-S5_Table_S1 [file lnaf013_suppl_supplementary_figures_s1-s5_table_s1.zip › Supplemental_Fig_S5.tif]
